# Vitamin D Prevents Endothelial Progenitor Cell Dysfunction Induced by Sera from Women with Preeclampsia or Conditioned Media from Hypoxic Placenta

**DOI:** 10.1371/journal.pone.0098527

**Published:** 2014-06-02

**Authors:** Lars Brodowski, Jennifer Burlakov, Ashley C. Myerski, Constantin S. von Kaisenberg, Magdalena Grundmann, Carl A. Hubel, Frauke von Versen-Höynck

**Affiliations:** 1 Department of Obstetrics and Gynecology, Hannover Medical School, Hannover, Germany; 2 Magee- Womens Research Institute and Foundation, University of Pittsburgh, Pittsburgh, Pennsylvania, United States of America; Queen’s University, Canada

## Abstract

**Context:**

Placenta-derived circulating factors contribute to the maternal endothelial dysfunction underlying preeclampsia. Endothelial colony forming cells (ECFC), a sub-population of endothelial progenitor cells (EPCs), are thought to be involved in vasculogenesis and endothelial repair. Low vitamin D concentrations are associated with an increased risk for preeclampsia.

**Objective:**

We hypothesized that the function of human fetal ECFCs in culture would be suppressed by exposure to preeclampsia-related factors–preeclampsia serum or hypoxic placental conditioned medium– in a fashion reversed by vitamin D.

**Design, Setting, Patients:**

ECFCs were isolated from cord blood of uncomplicated pregnancies and expanded in culture. Uncomplicated pregnancy villous placenta in explant culture were exposed to either 2% (hypoxic), 8% (normoxic) or 21% (hyperoxic) O_2_ for 48 h, after which the conditioned media (CM) was collected.

**Outcome Measures:**

ECFC tubule formation (Matrigel assay) and migration were examined in the presence of either maternal serum from preeclampsia cases or uncomplicated pregnancy controls, or pooled CM, in the presence or absence of 1,25(OH)_2_ vitamin D_3_.

**Results:**

1,25(OH)_2_ vitamin D_3_ reversed the adverse effects of preeclampsia serum or CM from hypoxic placenta on ECFCs capillary-tube formation and migration. Silencing of VDR expression by VDR siRNA, VDR blockade, or VEGF pathway blockade reduced ECFC functional abilities. Effects of VDR or VEGF blockade were partially prevented by vitamin D.

**Conclusion:**

Vitamin D promotes the capillary-like tubule formation and migration of ECFCs in culture, minimizing the negative effects of exposure to preeclampsia-related factors. Further evaluation of the role of vitamin D in ECFC regulation and preeclampsia is warranted.

## Introduction

Preeclampsia remains one of the most common causes of maternal and fetal morbidity and mortality in the developed world [1]–[3]. Although the pathogenesis of preeclampsia is still not fully understood, a multi-stage model is generally accepted. The utero-placental syndrome with impaired placental development in the first stage of the disease causes generalized maternal endothelial dysfunction as a main clinical feature of preeclampsia in the second stage [4]. An array of placenta-derived factors are candidate contributors to endothelial dysfunction in preeclampsia [5]–[7].

Endothelial progenitor cells (EPCs) are believed to play an important role in vascular homeostasis and in the repair of injured endothelium and neovascularization [8]. EPCs participate in both wound healing and angiogenesis. Decreased cell numbers of hematopoietic EPCs in the maternal circulation have been described as a potential sign of impaired endothelial repair capacity in preeclampsia [9], [10]. The late outgrowth sub-population of EPCs, also referred to as “endothelial colony forming cells” (ECFCs), have true endothelial-like characteristics, unlike the hematopoietic EPCs studied in the context of preeclampsia previously [11]. ECFCs are highly proliferative and migrate to sites of vessel formation, possessing the ability to differentiate into mature endothelial cells, to participate in vessel repair and to form *de novo* endothelium [12]. Recent data suggest that fetal ECFCs posses the ability to cross the placenta and participate in *de novo* maternal vessel formation in the pregnant uterus [13].

Vitamin D deficiency may be a risk factor for developing preeclampsia [14]–[18]. However, the underlying mechanisms are unclear. Our previous data suggest a VEGF dependent effect of vitamin D on ECFC proliferation and angiogenesis capability [19]. Given that the nature of the endothelial cell dysfunction and the role of ECFCs in preeclampsia are not entirely clear, we undertook this study in order to explore the effects of potentially relevant factors, i.e. serum from preeclamptic women or conditioned medium from placental villous explants exposed to hypoxic (2% O_2_) and hyperoxic (21%) oxygen tension, on ECFC function. In addition, we aimed to investigate whether the addition of 1,25(OH)_2_ vitamin D_3_ to the culture media can prevent ECFC dysfunction under these conditions.

## Materials and Methods

This collaborative study was performed at Magee-Womens Research Institute, Pittsburgh, PA and at the Department of Obstetrics and Gynecology, Hannover Medical School, Germany. The University of Pittsburgh Institutional Review Board and the Ethical Committee at Hannover Medical School approved the study and informed written consent was obtained from each woman.

### Patient Blood Sample Collection

Twelve healthy women with uncomplicated, normotensive pregnancies (controls) and 12 women with preeclampsia provided pre-delivery maternal blood samples for our study, 6 of each group being primiparous and 6 multiparous. All had singleton pregnancies. Clinical and demographic data describing these pregnant subjects, all of whom delivered at Magee-Womens Hospital, are presented in Table 1. Patients were matched for gestational age at the time of blood sampling, body mass index (BMI) and race. Patients with preeclampsia had gestational hypertension and proteinuria beginning after 20 weeks of pregnancy with resolution of clinical symptoms postpartum. Gestational hypertension was recognized as an absolute blood pressure ≥140 mmHg systolic and/or ≥90 mmHg diastolic after 20 weeks of gestation. Proteinuria was defined as ≥300 mg per 24-h urine collection, ≥2+ protein on voided urine sample, ≥1+ protein on catheterized urine specimen, or a protein-creatinine ratio of ≥0.3. Women with uncomplicated pregnancy were normotensive and without proteinuria throughout gestation, and delivered healthy babies. All patients were non-smokers by self-report, and were without clinical history of preexisting renal, vascular, or metabolic disease.

**Table 1:** Clinical and demographic data of patients who provided maternal blood samples.

Variable	Uncomplicated pregnancy (n = 12)	Preeclampsia(n = 12)	P value
Maternal age (y)	25.9±7.0	29.2±8.3	NS
Gestational age at time of blood sampling (wks)	36.5±4.2	37.4±2.3	NS
Gestational age at delivery (wks)	36.6±4.1	37.5±2.3	NS
Multiparous- n (%)	6 (50%)	6 (50%)	NS
Maternal pre-pregnancy BMI (kg/m^2^)	25.5±5.3	26.5±5.8	NS
Gestational SBP, pre-delivery (mm Hg)	120±13	151±17	<0.001
Gestational SBP before 20 week gestation (mm Hg)*	118±8	107±9	<0.01
Gestational DBP, pre-delivery (mm Hg)	71±7	91±9	<0.001
Gestational DBP before 20 week gestation (mm Hg)*	71±5	69±9	NS
Birth weight (g)	2877±930	2648±689	NS
Birth weight percentile	52.4±23.6	33.6±30.7	NS
Birth weight percentile <10^th^- n (%)	0 (0%)	2 (17%)	NS
Caesarean delivery- n (%)	3 (25%)	4 (33%)	NS
Labor at the time of blood sampling- n (%)	8 (67%)	6 (50%)	NS
Maternal Race, Black – n (%)	4 (33%)	4 (33%)	NS
Baby gender, male- n (%)	7 (58%)	3 (25%)	NS

BMI, body mass index; DBP, SBP, diastolic and systolic blood pressure (average of last three measurements). Data are given as mean ± SD or number (percentage). *Early BP values were not available for 3 preeclampsia patients; all patients were normotensive postpartum.

The maternal peripheral venous blood was withdrawn into sterile collection tubes. The blood samples were incubated at room temperature for 30 min and then centrifuged for 20 min at 2,000×g at RT. The serum was stored at −80°C for later use. Once all samples were acquired, four separate pools of serum were created (combining n = 6 patient samples/pool), namely primiparous uncomplicated pregnancy, multiparous uncomplicated pregnancy, primiparous preeclampsia (PE) and multiparous PE pools. Both the individual serum samples and the serum pools were used for the ECFC functional assays.

### Culture of Placental Villous Explants and Preparation of Conditioned Medium

The clinical and demographic data for women who provided both placentas and umbilical cord blood for this study are presented in Table 2. Placental villous explant preparation and culture was carried out according to published protocols with some modifications at Hannover Medical School [20]. Briefly, placentas from 16 uncomplicated, healthy pregnancies delivered by vaginal or Cesarean section were obtained within 10 min of delivery. All of these patients but two had singleton pregnancies. Biopsies were excised from the maternal side of the placenta, after removal of the decidua, midline between the central and lateral part of the placental edge. The tissue was immediately transported to the laboratory in ice-cold phosphate buffered saline (PBS) containing 2% penicillin/streptomycin. After rinsing the placental pieces in PBS to wash out blood, large vessels and decidua were removed by blunt dissection. Placental villous explants (1–2 mg each in size) were dissected and used for experiments under different oxygen conditions. An average of 50 mg of finely dissected villous tissue was placed into each well of a 12-well plate containing 1.5 ml of Medium 199 (Sigma-Aldrich, St- Louis, MO, USA) supplemented with 2% Fetal Bovine Serum (FBS, Biochrom, Berlin, Deutschland) and 1% penicillin–streptomycin (Biochrom, Berlin, Deutschland). The plates were incubated under controlled oxygen conditions (2% O_2_, 8% O_2_ and 21% O_2_) in three separate incubator chambers (Xvivo, Biospherix Inc., USA) at 37°C, 5% CO_2_ for 48 h. The CM was centrifuged (3,200 rpm, 4°C, 5 min) and the cell-free supernatants were frozen at −80°C for later use. For the experiments the CM were pooled according the oxygen conditions and stored in aliquots at −80°C. As control, M199 medium supplemented with 2% FBS, 1% penicillin–streptomycin (non-conditioned medium, NCM) was employed in the same ratio as conditioned medium (CM).

**Table 2:** Clinical and demographic data for the uncomplicated pregnant women who provided placental samples for villous explant culture (data are mean +/− SD).

Variable	
n	16
Maternal Age (y)	33±3.3
Multiparous – n (%)	9 (56%)
Maternal prepregnancy BMI (kg/m^2^)	27.1±6
Gestational SBP, pre-delivery (mmHg)	119.9±13.5
Gestational DBP, pre-delivery (mmHg)	68.6±11.3
Birth Weight (g)	3437.5±735
Gestational Age (weeks)	38.8±1.3
Caesarean delivery- n (%)	9 (56%)
Labor with delivery –n (%)	7 (44%)
Smoking- n (%)	0 (0%)

### ECFC Cell Isolation and Culture

Umbilical cord blood was collected into sterile Vacutainer tubes containing ethylenediaminetetraacetic acid (EDTA) immediately after delivery. Within 2 h of collection, the blood was centrifuged at 400 g for 10 min at room temperature, and the plasma was removed and replaced with plasma replacement buffer (PBS containing EDTA [7.4 g in 1 l, Sigma Aldrich, Steinheim, Germany] and 1% penicillin/streptomycin). The umbilical cord blood/plasma replacement buffer mixture was then doubled in volume by addition of an equal amount of isolation buffer (PBS, 2% FBS, 1% penicillin/streptomycin). This blood suspension was layered on Ficoll Plus (GE Healthcare, Buckinghamshire, England) and subjected to density gradient centrifugation (400 g, 40 min) with brake in the off position. The mononuclear cell layer was collected and 50 million cells per well seeded into collagen-1-coated 6-well plates (BD Biosciences, Heidelberg, Germany) using Endothelial Basal Medium 2 (EBM-2, Lonza, Walkersville, MD, USA), supplemented with EGM-2 Single Quot Kit (Lonza) in supplier-recommended concentrations of human recombinant epidermal growth factor, fibroblast growth factor, VEGF, ascorbic acid, hydrocortisone, recombinant insulin-like growth factor) containing 10% FBS and 1% penicillin/streptomycin. After 10–21 days (range) of cultivation ECFCs appeared as adherent single layers of cobblestone shaped, late outgrowth cells that formed colonies (>50 cells). Individual colonies were harvested using cloning rings and replated separately in tissue culture flasks. ECFCs were used at 80–90% confluence and between passages 4–7 in experiments.

### Phenotyping of ECFCs

The endothelial phenotype of the isolated blood cells was confirmed by flow cytometry using VEGFR-2, CD31, CD34, CD133 and CD45 as well as appropriate isotype controls, and by using fluorescein isothiocyanate-labeled Ulex europaeus agglutinin I (lectin; Sigma-Aldrich, Steinheim, Germany) for cell surface staining, and acetylated low-density lipoprotein (Dil-Ac-LDL; Biomedical Technologies, Stroughton, MA) to confirm cellular uptake of Dil-Ac-LDL, as described previously in detail [19].

### RNA Interference

RNA interference experiments were performed with siRNA for VDR (Silencer Validated siRNA VDR, Life Technologies, AM51331) and scrambled siRNA (Silencer Negative Control No. 1 siRNA, Life Technologies, Carlsbad, USA) using Dharmafect 1 (Dharmacon, Lafayette, CO, USA), according to the manufacturer’s instructions. Briefly, cells were plated and then transfected at 80–90% confluence with 50 nM siRNA for 24 h. The efficiency of siRNA transfection was tested using fluorescein-conjugated control siRNA.

### In vitro Angiogenesis Assay

We used an in vitro angiogenesis assay (endothelial tubule formation in Matrigel) as a test of ECFC function. 17,000 ECFCs/well were seeded into 96 well plates, each well pre-coated with 50 µl growth factor- reduced Matrigel (BD Biosciences, Bedford, MA). The ECFCs in Matrigel assay were exposed to pooled sera from normal pregnancy or preeclampsia patients (5% v/v concentration in EBM-2). We also tested the effect of the individual (non-pooled) serum samples (uncomplicated control versus preeclampsia; n = 12 samples each) at 5% v/v. The experiments were performed in the presence and absence of 10 nM 1,25(OH)_2_ vitamin D_3_ [21], [22]. In separate experiments, conditioned media (CM) or non-conditioned media (NCM) were added at 25% v/v concentration in EBM-2. After 14 h of incubation, digital images were obtained at 2.5x magnification. The total length of tubules per visual field (per well) was determined with Image J software. All experiments were done in triplicate wells from which values were averaged (n = number of experiments).

### Cell Migration Assay

To analyze ECFC migratory ability, ECFCs were pre-treated with EBM-2+2% v/v FBS, with or without 10 nM 1,25(OH)_2_ vitamin D_3_ for 24 h. The confluent ECFC monolayers were scratched using a sterile P200 pipette tip to produce a lane free of cells as described before [23]. Pooled sera from normal or preeclamptic patients was added at 5% v/v concentration. CM or NCM were added at 50% v/v concentration. The sera and media experiments were performed in the presence or absence of 10 nM 1,25(OH)_2_ vitamin D_3_. Light microscopic images were obtained immediately after the scratch (T_0_) and at the end of the experiment (18 h for CM; 8 h for serum). Migration into the scratch wound was analysed using Image J software and calculated as percentage of wound closure (percentage of original area that became occupied by cells by migration into the wound area). All experiments were done in quadruplicate wells from which values were averaged.

### VDR Silencing and VEGF Pathway Inhibition

The VDR was blocked with the VDR antagonist pyridoxal-5-phosphate (0.5 mM, Cell Signaling/New England Biolabs, Frankfurt am Main, Germany) or down-regulated by transfection with validated VDR siRNA. We and others have shown that vitamin D has a stimulatory effect on the VEGF pathway, which is important for angiogenesis and repair of vessels [19], [24]. Therefore, we also inhibited the VEGF pathway by SU5416 (0.5 µM, Sigma Aldrich, Steinheim, Germany).

### Vitamin D_3_ Measurement in Patient Serum

25(OH) Vitamin D concentrations in the individual maternal serum samples were determined using a LIAISON 25(OH) Vitamin D TOTAL Assay (DiaSorin Inc., USA), as per the manufacturers recommendations, at Hannover Medical School.

### Statistical Analysis

Statistical analysis was performed after testing for normality distribution by Kolmogorov-Smirnov-test. One-way analysis of variance, Kruskal-Wallis test, unpaired t-test, Mann-Whitney U or Wilcoxon-signed rank test were used as appropriate (Prism 4 software package, GraphPad Software Inc., La Jolla, CA). Demographic data are expressed as means and standard deviation (SD) and experimental data as means and standard error (SEM), with P<0.05 considered as statistically significant.

## Results

### Patient Demographics

Maternal age, pre-pregnancy body-mass index, race and parity were not statistically different between the preeclampsia cases and uncomplicated pregnancy controls who provided serum samples (Table 1). By definition, women with preeclampsia had significantly higher systolic and diastolic blood pressures at delivery compared to controls. The gestational age at the time of venipuncture was not significantly different between the groups. Table 2 shows the characteristics of the uncomplicated pregnancy patients that contributed their placentas for villous explant culture. Two of 16 were twin pregnancies.

### In vitro Angiogenesis

**Figure 1:**
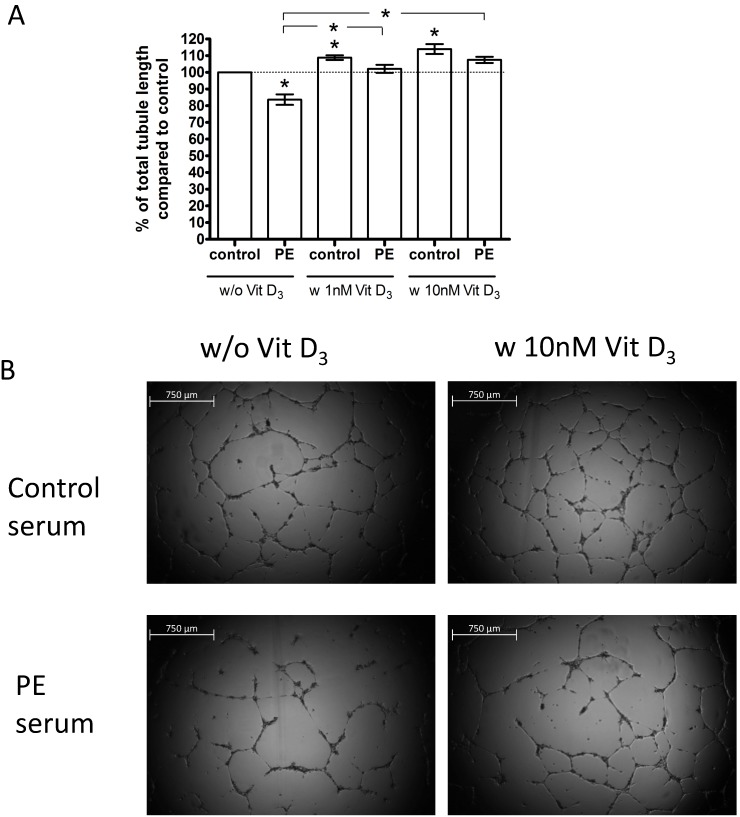
Effect of uncomplicated pregnancy (control) sera and preeclampsia (PE) sera, and 1,25(OH)_2_ vitamin D_3_, on capillary-tube formation by ECFCs in a Matrigel assay. ECFCs were cultured in endothelial basal medium (EBM) +5% v/v sera. Capillary-tube formation (average total tubule length per microscopic field) was analyzed after 14 h by visual microscopy at 2.5 magnification (A). Data are expressed as percentage of the control in the absence of vitamin D. Results represent mean values of total tubule length ± SEM of at least 6 independent experiments; *P<0.05 vs. control; Horizontal bars with asterisk (−*−): P<0.05, preeclampsia sera without vitamin D vs. preeclampsia sera with vitamin D. (B) Representative photomicrographs of ECFCs after incubation in Matrigel with EBM+5% v/v patient sera. Scale bar represents 750 µm.

Figures 1 A–B show the effects of serum from preeclampsia patients compared to serum from uncomplicated controls on capillary-tube formation by ECFCs, in which data from the primiparous serum pool and the multiparous serum pool are combined for each pregnancy outcome group. Here, tubule lengths are expressed as percent relative to the value (set at 100%) obtained in the presence of uncomplicated pregnancy pooled sera (control without supplemental vitamin D). Tubule formation was significantly less in the presence of preeclampsia serum (84±3%; P<0.001; n = 15 experiments) compared to uncomplicated pregnancy serum (Figure 1A). As shown in Figure S1, tubule assemblage was significantly impaired by either the primiparous preeclampsia pooled sera compared to primiparous control pooled sera (90±2.5%; P = 0.01; n = 6 experiments), or multiparous preeclampsia pooled sera compared to multiparous control pooled sera (79±4.5%; P = 0.01; n = 9 experiments).

We tested, the impact of 1,25(OH)_2_ vitamin D_3_ on tubule formation when in the simultaneous presence of pooled control or pooled preeclampsia sera. Vitamin D at 1 nM and 10 nM significantly increased total tubule length in the preeclamptic and control serum-treated groups (Figure 1A and Figure S1). As shown in Figure 1A, in which data from primiparous and multiparous sera are combined, 1 nM and 10 nM 1,25(OH)_2_ vitamin D_3_ stimulated tubule formation in the presence of both control sera (1 nM: 114±4%; P = 0.01; n = 15; 10 nM: 114±4%; P = 0.01; n = 12) and preeclampsia sera (1 nM: 124±4%; P = 0.01; n = 15; 10 nM: 123±4%; P<0.001; n = 12) such that the effects of preeclampsia vs. control sera were no longer significantly different (p>0.05).

We tested the effect of the individual (non-pooled) serum samples (uncomplicated control versus preeclampsia; n = 12 samples each) using one ECFC cell line derived from a control pregnancy and found comparable results. With control and preeclampsia serum samples tested as tandem pairs and matched for BMI, gestational and maternal age, 11 out of 12 serum samples from preeclamptic women lead to reduced tubule lengths compared to the control serum. In summary, serum of preeclamptic patients impaired angiogenetic capacity compared to control serum (87±4%; P = 0.01; n = 12). Relative to tubule formation in the presence of control pregnancy serum alone (100%), 1,25(OH)_2_ vitamin D_3_ increased tubule formation (1 nM: 106±4%; P = 0.02; n = 12; 10 nM: 110±3%; P = 0.01; n = 12). Relative to tubule formation in the presence of preeclampsia serum alone (100%), 1,25(OH)_2_ vitamin D_3_ likewise increased tubule formation (1 nM: 109±4%; P = 0.03; n = 12; 10 nM: 114±4%; P = 0.01; n = 12). In the presence of vitamin D (10 nM), preeclampsia vs. control patient sera no longer had significantly different effects (P>0.05).

**Figure 2:**
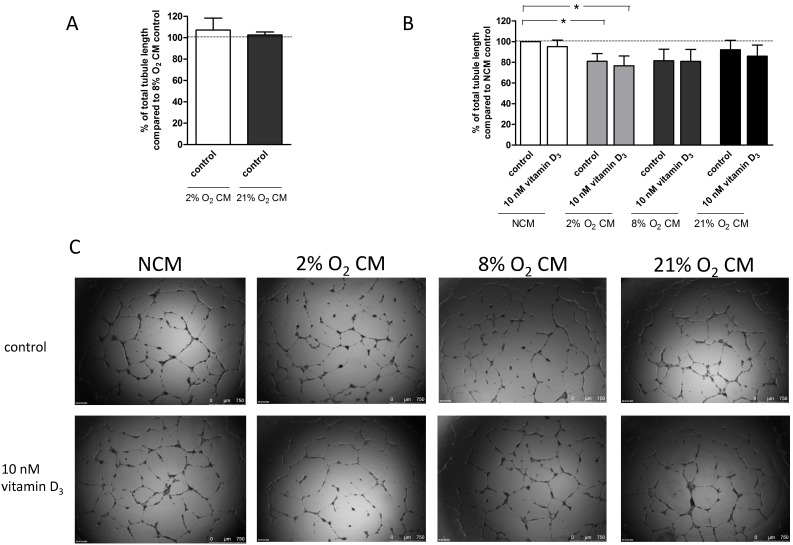
Effect of villous explant conditioned medium (2% O_2_, 8% O_2_, 21% O_2_ CM) and 1,25(OH)_2_ vitamin D_3_ on capillary-tube formation in a Matrigel assay. ECFCs were cultured in endothelial basal medium (EBM) +25% CM (A–C). Capillary-tube formation (average total tubule length per microscopic field) was analyzed after 14 h by visual microscopy at 2.5 magnification. Data are expressed as percentage of control (2A: 8% O_2_ CM+0 nM vitamin D_3_; 2B: NCM+0 nM vitamin D_3_). Results represent mean values of total tubule length ± SEM of at least 9 independent experiments; *P<0.05 vs. control, C: Representative photomicrographs of ECFCs after plating on Matrigel cultured in EBM+25% v/v CM. Scale bar represents 750 µm.

Figures 2 A–C display the results of treatment of ECFCs with placental villous explant CM (from cultures under 2%, 8% and 21% O_2_) compared to NCM. The effect of 1,25(OH)_2_ vitamin D_3_ is illustrated within the figures. ECFCs from uncomplicated pregnancies were used, and total tubule length was determined in the *in vitro* Matrigel angiogenesis model. There was no difference in tubule formation between the 2% O_2_, 8% O_2_ and 21% O_2_ CM (Figure 2 A). However, a reduction of angiogenesis with CM from 2% O_2_ villous explant culture was observed compared to NCM (81±7.4%; P = 0.03, n = 9). Vitamin D did not exhibit a significant effect under these conditions.

### Migration

**Figure 3:**
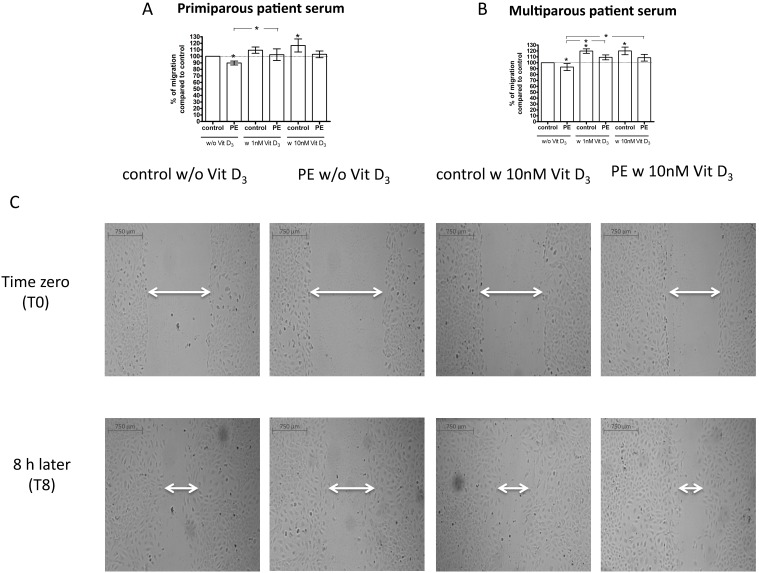
Effect of uncomplicated pregnancy (control) sera and preeclampsia (PE) sera from primiparous (A) and multiparous (B) women, and 1,25(OH)_2_ vitamin D_3_, on ECFC migration. ECFCs of uncomplicated pregnancies were cultured in endothelial basal medium (EBM) +5% v/v patient sera and treated with or without 1 nM or 10 nM 1,25(OH)_2_ vitamin D_3_. The migration of ECFCs into the scratch wound was assessed. ECFC migration was reduced in the presence of preeclampsia sera compared to control sera of primiparous (A) and multiparous (B) women. Vitamin D restored or improved migrative capacity. Results represent mean ± SEM percent wound filling of at least 6 independent experiments, *P<0.05 vs. control; Horizontal bars with asterisk (−*−): P<0.05, preeclampsia sera without vitamin D vs. preeclampsia sera with vitamin D. C: Representative images of ECFC monolayers with scratch wounds at 0 h (a, c) and 8 h (b, d) of incubation. Scale bar represents 750 µm.

As shown in Figure 3 A–C we found a reduction in migration of ECFCs when in the presence of primiparous (90±3%; P = 0.02; n = 6 experiments) or multiparous (92±6%; P = 0.03; n = 12 experiments) preeclampsia pooled sera compared to the corresponding control pooled sera. Combining data from primi- and multiparous patient samples, the negative effect of preeclampsia pooled sera on ECFC migration remained significant (92±4%; P = 0.01; n = 18).

As shown in Figure 3, the simultaneous addition of 1,25(OH)_2_ vitamin D_3_ increased the migrative abilities of ECFCs into the scratch wound during incubation with pooled primiparous (1 nM: 114±10%; P = 0.03; n = 6; 10 nM: 115±6%; P = 0.053; n = 6) or multiparous (1 nM: 120±5%; P = 0.01; n = 12; 10 nM: 114±4%; P = 0.01; n = 9) preeclampsia pooled sera (100%). We found a stimulating effect of 10 nM 1,25(OH)_2_ vitamin D_3_ in the presence of pooled sera from primiparous controls (1 nM: 109±5%; P = 0.11; n = 6; 10 nM: 117±10%; P = 0.03; n = 6) and stimulating effect of both 1 nM and 10 nM 1,25(OH)_2_ vitamin D_3_ in the presence of pooled sera from multiparous controls (1 nM: 120±4%; P<0.001; n = 12; 10 nM: 120±6%; P = 0.03; n = 9). When combining data from primi- and multiparous patient pools, 1,25(OH)_2_ vitamin D_3_ enhanced ECFC migration both in the presence of control sera (1 nM: 116±3%; p<0.001; n = 18; 10 nM: 119±5%; P = 0.01; n = 15) and preeclampsia sera (1 nM: 118±5%; P = 0.01; n = 18; 10 nM: 115±3%; P<0.001; n = 15), such that the differential effects of case vs. control sera remained significant. ECFC migration in the presence of preeclampsia sera + vitamin D was not different from uncomplicated pregnancy sera alone.

**Figure 4:**
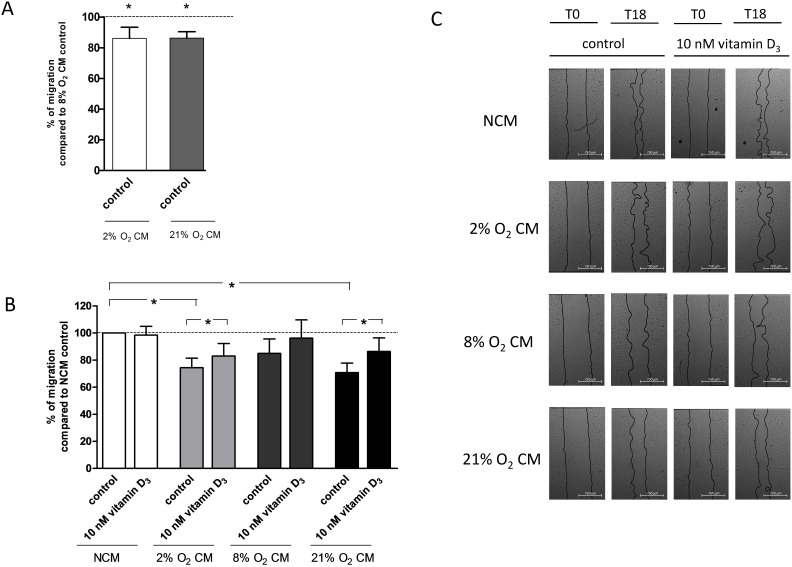
Effect of villous explant conditioned medium (2% O_2_, 8% O_2_, 21% O_2_ CM) and 1,25(OH)_2_ vitamin D_3_ on ECFC migration. ECFCs of uncomplicated pregnancies were cultured in endothelial basal medium (EBM) +50% v/v CM and treated without (vehicle control) or with 10 nM 1,25(OH)_2_ vitamin D_3_. The migration of ECFCs into the scratch wound was assessed. ECFC migration was lower in 2% O_2_ and 21% O_2_ villous explant CM compared to 8% O_2_ CM (A) and NCM (B). Vitamin D restored migrative capacity. Results represent mean ± SEM percent wound filling, N = 10, *P<0.05 vs. control (0 nM vitamin D). C: Representative images of ECFC monolayers with scratch wounds at 0 h and 18 h of incubation. Scale bar represents 750 µm.

Placental villous explant CM derived from 2% O_2_ incubations (86.1±7.2%, P = 0.02, n = 9) and 21% O_2_ incubations (86±4.1%, P = 0.02, n = 10) significantly reduced ECFC migration, when compared to CM from 8% O_2_ (Figure 4 A). Compared to NCM, explant CM from 2% O_2_ (74±7%; P = 0.005) and 21% O_2_ (71±7.1%, P = 0.003, n = 10), but not 8% O_2_, impaired ECFC migration (Figure 4 B). Compared to absence of vitamin D, 1,25(OH)_2_ vitamin D_3_ improved endothelial cell migration in 2% O_2_ CM (110±4.4%, P = 0.046) and 21% O_2_ CM (124±8.7%, P = 0.02), but not significantly in 8% O_2_, (113±7.9%, P = 0.15) (n = 10). 1,25(OH)_2_ vitamin D_3_ normalized the negative effects of 2% O_2_ CM (95±5.5%, P = 0.36) and 21% O_2_ CM (108±9.9%, P = 0.46) when compared to 8% O_2_ CM (data not shown). ECFCs treated with 2%, 8% or 21% CM and vitamin D were also not statistically different compared to NCM (P>0.05), (Figures 4 B and C).

### VDR Blocking and Inhibition of VEGF Pathway

**Figure 5:**
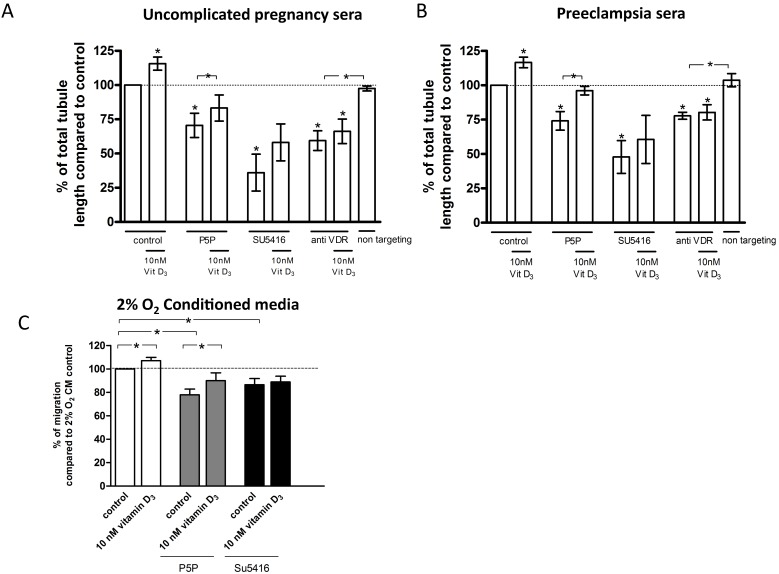
Effect of 1,25(OH)_2_ vitamin D_3_ and the inhibitors pyridoxal-5-phosphate, SU5416 and vitamin D receptor (VDR) small interfering (si)RNA on ECFC capillary-tube formation or migration. ECFCs were incubated with uncomplicated pregnancy sera (A) or preeclampsia sera (B) or 2% O_2_ villous explant conditioned medium (C), and in the presence or absence (control) of 0.5 mM pyridoxal-5-phophate, 0.5 µM SU5416, VDR siRNA, or non-targeting (scrambled) siRNA. The effects of each of these exposures were additionally examined both in the absence (vehicle) or presence of 10 nM 1,25(OH)_2_ vitamin D_3_. Total tubule lengths (A, B) or migration (C) were determined and expressed as percent relative to control. Results represent mean ± SEM of at least 4 independent experiments. *P<0.05 compared to control. Other significant differences (P<0.05) are indicated by the horizontal bars.

The effects of VDR and VEGF inhibitors (P5P and SU4516, respectively) on ECFC tubule formation were tested in the presence of pooled control sera (Figure 5 A) or pooled preeclampsia sera (Figure 5 B). P5P inhibited tubule formation (percent relative to control sera alone: 71±8.9%; P = 0.045; n = 4; percent relative to preeclampsia sera alone: 74±6.8%; P = 0.02; n = 5). SU5416 similarly inhibited tubule formation (percent relative to control sera alone: 36±13.5%; P = 0.02; n = 4; percent relative to preeclampsia sera alone: 48±11.9%; P = 0.02; n = 4). Silencing of the VDR also impaired tubule formation (percent relative to control sera alone: 59±7.2%; P = 0.01; n = 4; percent relative to preeclampsia sera alone: 78±2.6%; P = 0.01; n = 4), (data compared to non-silenced control ECFCs). Non-targeting siRNA transfected ECFCs were used as an internal control. Total ECFC tubule lengths were not affected by the non-targeting siRNA (percent relative to control sera alone: 98±1.8%; P = 0.27; n = 4; percent relative to preeclampsia sera alone: 104±4.8%; P = 0.5; n = 4). Vitamin D significantly increased tubule formation in the presence of uncomplicated pregnancy (Figure 5 A) or preeclampsia sera (Figure 5 B) (control = sera without VDR or VEGF inhibitors). Vitamin D significantly reversed the negative effects of VDR blockade (P5P) on tubule formation, and non-significantly attenuated the negative effects of VEGF blockade (SU5416), but had no effect after VDR silencing (Figure 5 A and B).

The impact of VDR or VEGF pathway inhibition on ECFC migration was tested in the presence of 2% O_2_ incubation-derived villous explant culture CM (Figure 5 C). A significant reduction of scratch wound closure was observed in the presence of P5P (78±4.9%; P = 0.004, n = 7) and SU5416 (87±5.3%; P = 0.04, n = 8). Vitamin D had a rescuing effect on ECFC migration after blocking the VDR (P5P+VD: 111±4.4%; P = 0.049, n = 7), but not the VEGF pathway (SU5416+ VD: 104±4.6%; P = 0.46, n = 8).

### Maternal Vitamin D Status

None of the patients had normal (replete) vitamin D levels (>30 ng/ml [25]). More women with preeclampsia (PE: 7/12, NP: 3/12) showed severe vitamin D deficiency (<10 ng/ml), although not significantly so (P = 0.21). There was no significant relationship between maternal serum vitamin D concentration and fetal ECFC response to the patient serum in cell culture (P = 0.45).

## Discussion

One major finding of this study is that ECFCs exhibited markedly decreased tubule formation and migration *in vitro* in response to serum from women with preeclampsia compared to serum from women with uncomplicated pregnancies. Both pooled serum and individual patient serum samples from women with preeclampsia exhibited these inhibitory effects. A second major finding is that suppression of ECFC migration was also observed in response to conditioned media collected from placental villous explants cultured under aberrant oxygen conditions [2% O_2_ (“hypoxic”) or 21% (“hyperoxic”)] when compared to either 8% O_2_ (“normoxic”) or non-conditioned media. These inhibitory effects on ECFC behaviors were substantially reversed by exogenous administration of vitamin D in the physiologic range. VDR signaling antagonism (pyridoxal-5-phosphate) reduced ECFC tubule formation and migration in a fashion partially reversed by exogenous vitamin D, likely related to competition for binding to the vitamin D receptor (VDR).

During the first weeks of normal pregnancy, the placental environment is hypoxic (∼2% O_2_), but the oxygen level rises up to 6–8% O_2_ around 12 weeks of gestation, which is considered physiologic during the second and third trimester of pregnancy [26]. At term, 6–8% O_2_ is thought to represent “normoxia” and 1–2% O_2_ “hypoxia” for villous tissue [27]. In this study 2% O_2_ was used as hypoxic, 8% O_2_ as normoxic and 21% O_2_ as “hyperoxic” conditions as in previous studies [28], [29]. We speculate that our findings of reduced ECFC functional capacities during incubation with both low (2%) and high (21%) O_2_ might relate to increased generation of anti-angiogenic/pathogenic factors under these conditions compared to 8% O_2_ explant culture conditions. Both hypoxia, especially with fluctuations in oxygen concentration, and hyperoxia can result in placental oxidative stress with release of inflammatory cytokines and/or anti-angiogenic factors [30]. In preeclampsia, low or fluctuating oxygen levels might persist due to impaired utero-placental blood flow and lead to disturbances in the placenta. The associated increased release of placenta-derived factors is believed to contribute to the disturbed maternal endothelial homeostasis [1], [31]. The nature/effects of the circulating placental factors remain intensively investigated [31].

Previously, metabolic footprinting of placental villous explant conditioned culture media identified differences as a function of normoxic, hypoxic and hyperoxic incubation conditions [32]. Uncomplicated pregnancy villous tissue incubated under hypoxic conditions, and preeclampsia villous tissue incubated under normoxic conditions produced very similar metabolic footprints [32]. Others have reported that endothelial cell proliferation was reduced by plasma, but not serum, from preeclamptic women [33]. This is in contrast to our study were we show a significant negative effect of preeclampsia serum on ECFCs in vitro.

Vitamin D deficiency has been linked to an approximate 5-fold increased risk for the development of preeclampsia [14]–[16]. The vitamin D receptor (VDR) is expressed in human placenta, endothelial cells and (we have shown) in cord ECFCs [19], [34], [35]. Potentially relevant to preeclampsia, vitamin D regulates key target genes associated with implantation, trophoblast invasion and anti-inflammatory responses in maternal decidua and fetal trophoblast [36]–[38]. Vitamin D regulates angiogenesis through direct effects on VEGF gene transcription [19], [24], [39]. Our published data show a stimulating and VEGF-dependent effect of physiologic concentrations of vitamin D on fetal (cord blood) ECFC function [19]. We now demonstrate a stimulating effect of vitamin D on ECFC migration and capillary-tube formation, overcoming adverse effects of serum from preeclamptic women. Vitamin D also enhanced ECFC function after treatment with hypoxic or hyperoxic villous CM. These effects were dependent on VDR activation as indicated by silencing and blocking the receptor. Here we also confirmed that blocking of the VEGF pathway impairs ECFC function. However, vitamin D did not substantially restore the inhibiting effect of the VEGF pathway inhibitor SU5416 in the presence of patient sera or villous CM.

Vitamin D was able to rescue the angiogenic deficits caused by preeclampsia sera (Figure 1), but not those caused by 2% oxygen conditioned media (Figure 2). Chronic hypoxia might not exactly model the effects of fluctuations (hypoxia-reoxygenation) thought to frequently occur with preeclampsia [30]. The milieu in maternal serum may be more complex, with levels of maternally derived factors differing by pregnancy outcome group. Alternatively, concentrations of anti-angiogenic factors may be higher in placenta CM compared to maternal serum. The magnitude of change (increase) in tubule formation effected by vitamin D was greater when in the presence of preeclampsia sera compared to uncomplicated pregnancy sera, such that tubule formation became equalized (Figure 1). This differential response to vitamin D according to sera was not observed with migration. The reason for this non-uniformity of response remains unclear.

Preeclampsia is a risk factor for cardiovascular events in the mother and offspring later in life [31], [40]. Potentially germane to this, early life vitamin D deficiency in a rat model was associated with endothelial dysfunction and elevated blood pressure in the offspring [41], and a study in mice found vitamin D deficiency in pregnancy to lead to maternal hypertension and altered placental and fetal development [42]. These *in vivo* data are consistent with the hypothesis that vitamin D insufficiency predisposes pregnant women and their offspring to disturbed endothelial homeostasis.

ECFCs, a sub-population of EPCs, posses the unique ability for vasculogenesis – the *de novo* formation of blood vessels from progenitor cells. The few studies of fetal ECFCs and pregnancy complications describe a reduction in fetal ECFC numbers and colony formation in diabetic pregnancies or pregnancies with growth restricted infants [43], [44]. Most interestingly recent data point to the ability of human fetal ECFCs, but not human fetal endothelial cells, to transmigrate to the maternal bloodstream and then home to locations of maternal uterine vasculogenesis [13]. Our data suggest that fetal ECFCs respond to pathogenic stimuli by impairment of functional activities and that these can be reversed by vitamin D. A logical future step would be to accumulate conditioned media from gestational age-matched preeclampsia and uncomplicated pregnancy villous placenta for comparison. The extent to which villous fragments in explant culture accurately retain their *in vivo* phenotype is uncertain. However, it was previously reported that, compared to conditioned media from uncomplicated, term pregnancy villous explants, conditioned media from preeclampsia placental villous explants suppress *in vitro* tubule formation and migration of human umbilical vein endothelial cells (HUVEC) in culture [45]. These effects appeared to be attributable to increased secretion of the soluble receptor, soluble vascular endothelial growth factor receptor-1 (sVEGFR-1, also known as sFLT1), by preeclampsia explants, consistent with the elevated placental secretion of sFLT1 in *vivo* [5].

Several reports indicate that 1,25(OH)_2_ vitamin D either decreases or has no effect on endothelial cell proliferation or angiogenesis *in vivo* or *in vitro* [46], [47]. The divergent results might reflect heterogeneity among endothelial cell subtypes. ECFCs reportedly differ from mature human umbilical vein endothelial cells (HUVEC) or human umbilical artery endothelial cells in the expression of differentiation-related surface markers, proliferation rates, or telomerase activities [48]. However the reason for the apparent distinct proangiogenic effects of vitamin D on ECFCs is presently unclear. To speculate, vitamin D might upregulate the release of factors by progenitor cells that, in turn, stimulate angiogenic behaviors in autocrine fashion. Hematopoietic endothelial progenitor cells, a more prevalent circulating cell type compared to ECFCs, express functional vitamin D receptors. Conditioned media from vitamin D treated hematopoietic endothelial progenitors increased tubule networks of human aortic endothelial cells in Matrigel in vitro, whereas vitamin D alone did not [49].

To our knowledge this is the first study to investigate the effect of placenta-derived factors on ECFC function and to explore a putative positive role of vitamin D for restoring endothelial function in this context. Effective preventive or therapeutic strategies for preeclampsia do not exist to date. It is plausible that ensuring vitamin D sufficiency before and during pregnancy will reduce endothelial dysfunction and disease development in mother and offspring.

## Supporting Information

Figure S1 **Effect of uncomplicated pregnancy (control) pooled sera and preeclampsia (PE) pooled sera from primiparous (A) and multiparous (B) women, and 1,25(OH)_2_ vitamin D_3_, on capillary-tube formation by ECFCs in a Matrigel assay.** ECFCs were cultured in endothelial basal medium (EBM) +5% v/v sera. Capillary-tube formation (average total tubule length per microscopic field) was analyzed after 14 h by visual microscopy at 25x magnification. Data are expressed as percentage of the control in the absence of vitamin D. Results represent mean values of total tubule length ± SEM of at least 6 independent experiments; *P<0.05 vs. control; Horizontal bars with asterisk (−*−): *P<0.05*, preeclampsia serum without vitamin D vs. preeclampsia serum with vitamin D.
(TIFF)
