# Supplementary figures and images for: Vitamin D Prevents Endothelial Progenitor Cell Dysfunction Induced by Sera from Women with Preeclampsia or Conditioned Media from Hypoxic Placenta
Source: PLoS One. 2014 Jun 2;9(6):e98527. doi: 10.1371/journal.pone.0098527 (PMC4041729; doi:10.1371/journal.pone.0098527)

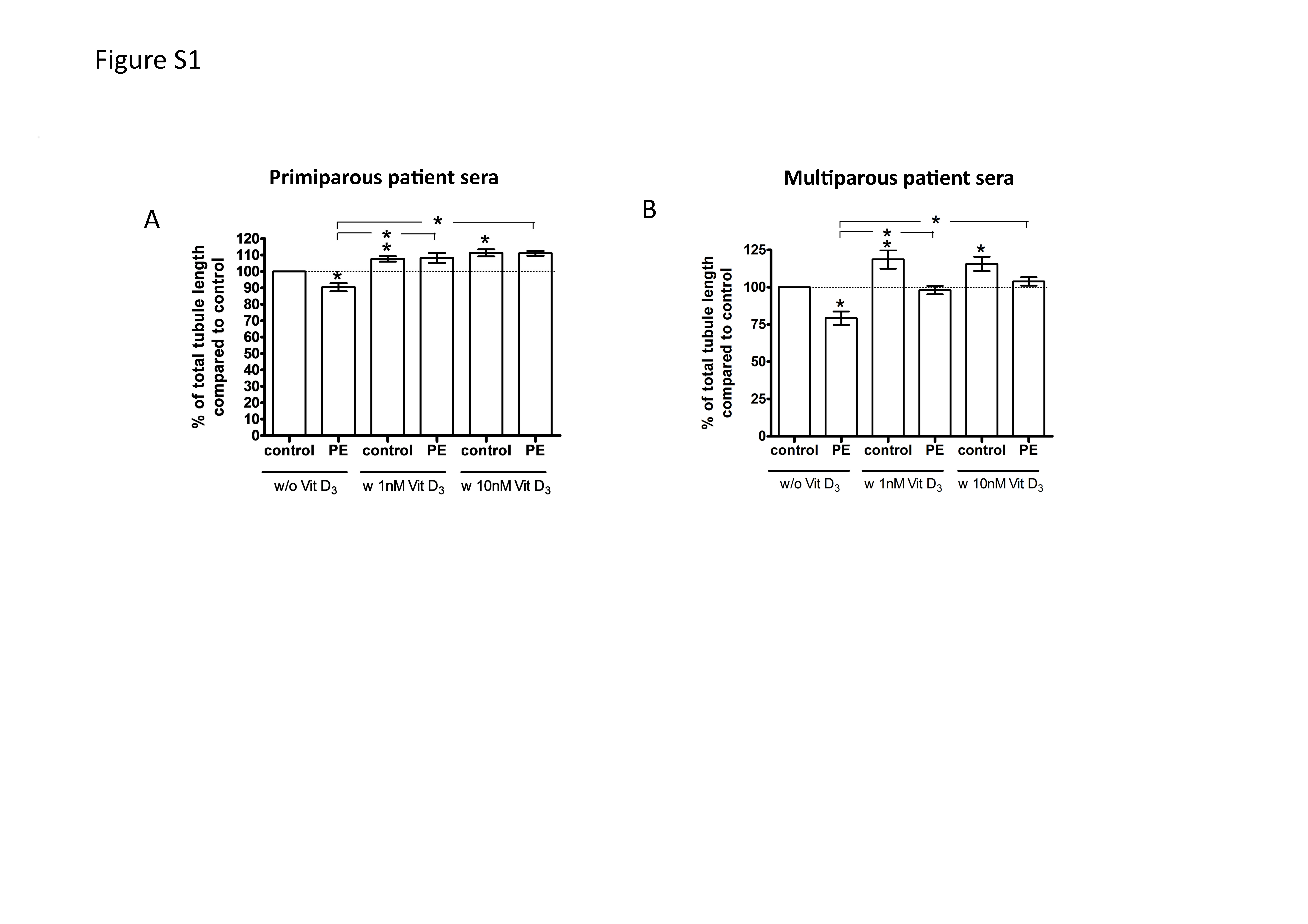

Supplement: Figure S1 — Effect of uncomplicated pregnancy (control) pooled sera and preeclampsia (PE) pooled sera from primiparous (A) and multiparous (B) women, and 1,25(OH)2 vitamin D3, on capillary-tube formation by ECFCs in a Matrigel assay. ECFCs were cultured in endothelial basal medium (EBM) +5% v/v sera. Capillary-tube formation (average total tubule length per microscopic field) was analyzed after 14 h by visual microscopy at 25x magnification. Data are expressed as percentage of the control in the absence of vitamin D. Results represent mean values of total tubule length ± SEM of at least 6 independent experiments; *P<0.05 vs. control; Horizontal bars with asterisk (−*−): P<0.05, preeclampsia serum without vitamin D vs. preeclampsia serum with vitamin D. (TIFF) [file pone.0098527.s001.tif]
